# Quantifying the Mechanical Properties of Materials and the Process of Elastic-Plastic Deformation under External Stress on Material

**DOI:** 10.3390/ma8115385

**Published:** 2015-11-03

**Authors:** Jan Valíček, Marta Harničárová, Andreas Öchsner, Zuzana Hutyrová, Milena Kušnerová, Hakan Tozan, Vít Michenka, Vladimír Šepelák, Dušan Mitaľ, Jozef Zajac

**Affiliations:** 1 Institute of Physics, Faculty of Mining and Geology, VŠB—Technical University of Ostrava, 708 33 Ostrava, Czech Republic; marta.harnicarova@vsb.cz (M.H.); milena.kusnerova@vsb.cz; (M.K.); htozan@dho.edu.tr (H.T.); vladimir.sepelak@vsb.cz (V.Š.); 2 Institute of Clean Technologies for Mining and Utilization of Raw Materials for Energy Use, Faculty of Mining and Geology, VŠB—Technical University of Ostrava, 708 33 Ostrava, Czech Republic; 3 RMTVC, Faculty of Metallurgy and Materials Engineering, VŠB—Technical University of Ostrava, 708 33 Ostrava, Czech Republic; 4 Nanotechnology Centre, VŠB—Technical University of Ostrava, 708 33 Ostrava, Czech Republic;; 5 Griffith School of Engineering, Griffith University, Southport Queensland 4214, Australia; oechsner@griffith.edu.au; 6 Faculty of Manufacturing Technologies of TUKE with a seat in Prešov, Prešov 080 01, Slovakia; dusan.mital@tuke.sk (D.M.); jozef.zajac@tuke.sk (J.Z.); zuzana.hutyrova@tuke.sk (Z.H.); 7 Turkish Naval Academy, 34942 Istanbul, Turkey; 8 Laboratory of Mechanical Properties, VÚHŽ, 739 51 Dobrá, Czech Republic; michenka@vuhz.cz; 9 Institute of Nanotechnology, Karlsruhe Institute of Technology, 76344 Eggenstein-Leopoldshafen, Germany; vladimir.sepelak@kit.edu

**Keywords:** surface topography, mechanical equivalents, plasticity, abrasive waterjet cutting, deformation

## Abstract

The paper solves the problem of the nonexistence of a new method for calculation of dynamics of stress-deformation states of deformation tool-material systems including the construction of stress-strain diagrams. The presented solution focuses on explaining the mechanical behavior of materials after cutting by abrasive waterjet technology (AWJ), especially from the point of view of generated surface topography. AWJ is a flexible tool accurately responding to the mechanical resistance of the material according to the accurately determined shape and roughness of machined surfaces. From the surface topography, it is possible to resolve the transition from ideally elastic to quasi-elastic and plastic stress-strain states. For detecting the surface structure, an optical profilometer was used. Based on the analysis of experimental measurements and the results of analytical studies, a mathematical-physical model was created and an exact method of acquiring the equivalents of mechanical parameters from the topography of surfaces generated by abrasive waterjet cutting and external stress in general was determined. The results of the new approach to the construction of stress-strain diagrams are presented. The calculated values agreed very well with those obtained by a certified laboratory VÚHŽ.

## 1. Introduction

Knowledge in the area of material properties has been obtained over a very long period of time. The current state of the problem is causally associated with and is a logical continuation of this historical development. At present, it is potentiated by the fast development of technology and the requirements given by the properties of the materials being worked with. The main aspect is the level of dimensioning of structural elements from the point of view of their functional load, safety, life, and also costs and environmental impacts. These are also observed parameters of technological products, *i.e.*, machines, constructions and structures from the point of view of their competitiveness. What has to suit the rapid development of technology is the sufficiently fast development of measurements of physico-mechanical properties of exploited materials, because the measured properties are the primary and fundamental tools of the designer to satisfy the requirements given. Moreover, it can be stated that, in some aspects, the development of measurement methods somehow falls behind the development of technology and requirements for engineering design quality. By means of classical methods, it is difficult to obtain a number of very important physico-mechanical parameters with the required accuracy. It is the case of, for example, sufficiently accurate determination of engineering and true Young’s modulus, elastic limit, yield point and ultimate strength, including the still existing uncertainty in measurement and also in the analytical processing of the stress–strain relationship in the plastic area of material deformation [1,2]. All these parameters are of highest importance to the safety, stability and life of constructions, structures and machines. There are several methods and models developed to characterize the deformation behavior of materials: tensile tests, compression tests, torsion tests, *etc.* [3,4,5]. These standard tests are very laborious, tedious, and technically and financially demanding processes. It should be emphasized that these tests are carried out in different laboratories with different subjective and objective conditions. Therefore, the results obtained for the same materials are often incomparable, and in many cases, tabulated values usually have large variances and are taken to be indicative only, and therefore, cannot be considered as a proven value for a specific material.

The mechanical properties are used to describe particular parameters quantifying the resistance of a material to deformation and failure [6,7,8,9,10,11]. Current scientific databases contain a large number of papers in the fields of modeling and simulation of the plastic deformation of materials under specific types of loading. Of particular importance are: continuum-based models of improved anisotropic plasticity models, dynamic dislocation simulation of microstructure and plasticity and mainly simulation of the plastic deformation of materials in more realistic deformation conditions, discrete dislocation statics and dynamics, lattice defects at the grain scale, and poly-crystal plasticity deformation [12,13,14,15]. These models describe the material response to deformation across the whole fundamental length scales, from the atomic to the continuum scale. Different methods of surface analysis are described in the world literature, but only from the point of view of surface geometry, and not from point of view of analysis of stress-deformation material behaviour.

The aim of this paper is to introduce, in addition to the technological aspects, a new analytical evaluation procedure for specific elements of the topography of surfaces generated by flexible machining tools. The structure as well as the surface topology is a unique representation of the physical-mechanical reaction of a material subject to the cutting tool. As far as quality is concerned, this aspect is generally accepted; nevertheless, neither quantified analytical processing, nor description of it has been carried out yet. Here, a method of deriving several basic physical-mechanical properties and their equivalents will be derived on the principles of generating machined surface structures and textures using a flexible tool. Issues of material behavior at the stress level of the elastic limit, *i.e.*, in elastic-plastic and plastic areas, have never been adequately solved at the level of applied and fundamental research. The accomplishment of the main objective and concept of this paper leads to a new hypothesis for creating stress-strain diagrams. The idea is to see what role surface topography can play in identifying and predicting the deformation stress state of different materials including the determination of equivalents of mechanical parameters. The intensity of plasticity of material under external stress, the determination of the so-called plasticity index, and its use for the construction of stress-strain curves and plasticity growth rates are discussed in detail in Section 4.3.

## 2. Current State of Knowledge in the Area of Abrasive Waterjet Cutting

The majority of experimental and theoretical works focus on the presentation of authors’ own results, but separately, without deep analysis and context. It is the material itself, not being adequately addressed yet, that plays an important role in cutting. That is why the concept of research in the work concerned focuses on the mechanical properties of the cut material. Based on the analysis of available sources in the area of abrasive waterjet cutting, it can be critically stated that insufficient attention is paid to the topography of final surfaces. Structural and textural elements of cut wall topography are, on the contrary, in the concept of the submitted method of solving, the basic source of substantial research information on the mechanism of primary contact between the material and the disintegration tool [16,17,18,19,20]. Thus, one can agree with the opinion that the mechanism of abrasive waterjet cutting utilizing a mechanically flexible tool and being affected by many other factors entering the process of material disintegration is, from the point of view of analytical approximation, elaboration and description, complicated. For this reason, these issues have not been solved by systematic and comprehensive procedures yet. That is why adequate possibilities for an exact approach to the design and optimization of technological parameters are still missing in operating practice and in development of the technology.

The knowledge of the topography function is of high importance to other analyses and prediction of the state of surface topography with a change in the cut material or technological regime as well as at the feedback control of final surface roughness. The analytical knowledge of rules of the zonal distribution of values of geometrical and stress-strain parameters is a precondition for the development of prediction equations that are important not only for the selection of a suitable technology. Reasons for the insufficient level of existing knowledge are given in the following points:

- Insufficient examination of the physical-mechanical parameters of materials with the absence of uniform mathematical formulations,
- Insufficient examination of the physical-mechanical and stress-strain integrity of the system: technological parameters—tool, material, or state—of surface topography,
- Insufficient utilization of rules of distribution and zonality of geometrical parameters of surfaces generated by abrasive waterjet cutting,
- Inconsistency in the interpretation of achieved results,
- Conducting research mostly on cuts with an insufficient depth,
- Outstanding timing of performance parameters of the process,
- Absence of sufficiently generalized theoretical prediction of limit depths achieved in different materials, roughness and behavior of main deformation functions in relation to the technological parameters of cutting,
- Shortage of suitably conceived mathematical models for designing the technology,
- Existing neglect of specific elements of surface topography suitable for wide use in theory and practice.

## 3. Experimental Section

### 3.1. Experimental Setting of Abrasive Waterjet Technology

Thirty samples with a size of 20 mm × 20 mm were prepared by abrasive waterjet cutting (from various metal materials, such as steel, aluminium, brass, and others). The surface irregularity distribution was measured using the optical profilometer MicroProfFRT. The used materials were: STN 17 251/AISI 309, STN 17 240/AISI 304, STN11523/S355J2G3, STN 15 230/DIN 30CrV9, STN 12024/AISI 1020, and others. The measurements were made using the conventional contact profilometer on 22 measurement levels and 4 sides created at different traverse speeds of the cutting head. As the abrasive material, recycled Garnet 80 MESH was used. All main cutting parameters were determined in advance for each material individually in compliance with the procedure patented by the authors according to [21]. For further analyses, the samples were available for measurement using the optical profilometer.

### 3.2. Measurement of Surface Roughness

**Figure 1:**
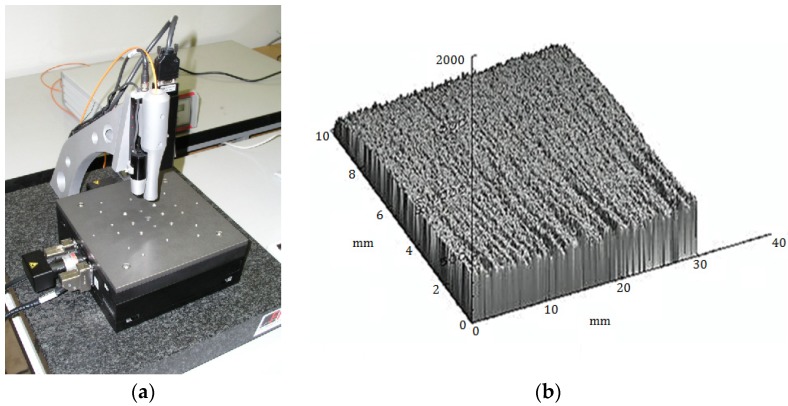
(**a**) General view of the optical profilometer FRT MicroProf produced by FRT; (**b**) 3 D image of cutting wall.

We measured the three-dimensional surface topography using an optical profilometer FRT MicroProf (Fries Research & Technology GmbH (FRT), Bergisch Gladbach, Germany). On the basis of these measurements (see Figure 1a,b), data were analyzed and interpreted to describe theoretically the topography of the surface.

Using a CHR 150 optical sensor, the sample is illuminated by focused white light. The internal passive optics, using chromatic aberration, splits the white light into different colors. A spectrometer detects the color of the light reflected by the sample and determines the position of the focus point, and by means of an internal calibration table, the vertical position measured on the sample surface. The optical sensor is non-movable, the sample under study lies on a scanning table. The same optical fibre collects scattered light from the surface under study. This light is analysed by means of the spectrometer. Results of the measurement have the form of a vector and/or the matrix of heights of the surface irregularities. The basic parameters of FRT MicroProf are as follows: *xy* minimum range: 200 × 200 μm^2^, *xy* maximum range: (100 × 100) 10^−6^ μm^2^, measurement range: 300 μm–3 × 10^3^ μm, vertical resolution: 3 × 10^−3^ μm, lateral resolution: 2 μm, maximum angle of inclination of the surface roughness to the mean plane: 30 [22,23,24].

### 3.3. Tensile Testing

**Figure 2:**
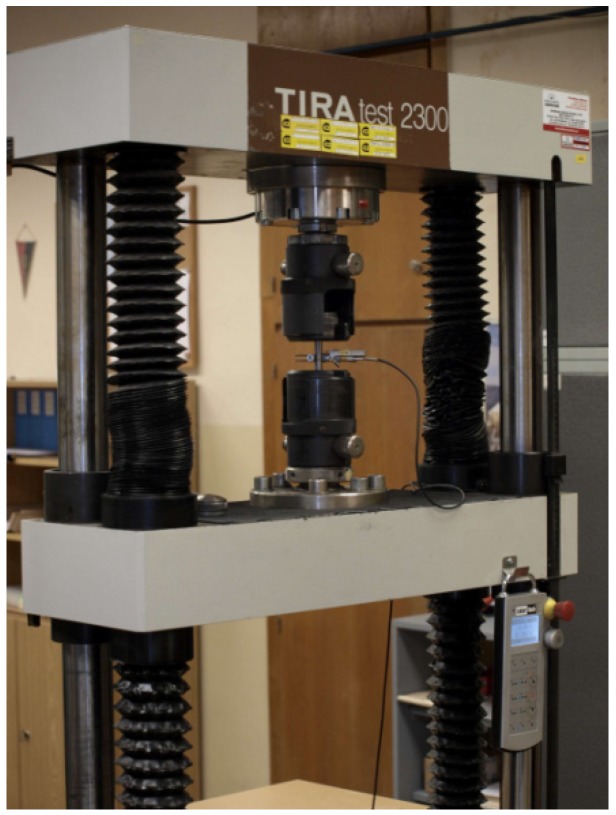
Universal testing machine—Tira Test 2300.

Tensile test was carried out in accordance with ISO 6892-1. Round shaped test-pieces with threaded ends (M12) of dimensions: diameter of testing sample *d* = 8.0 mm, gauge length *L_c_* = 45 mm, total length *L_t_* = 85 mm were used for testing. The test-pieces were conditioned at +22 °C/40% r. h. for 6 h prior to the test. Used test rate: 20 MPa/s up to achieve yield strength, 0.004 s-1over yield strength up to final breakage of the test-piece. A piezo-electric extensometer was used for the determination of the yield strength. All the testing equipment was calibrated in accordance with ISO 7500. Testing was performed on a Tira Test 2300 universal testing machine (Figure 2).

## 4. Problem Solving

The problem concerns a fundamentally new method of determination of equivalents of mechanical material parameters from the topography of surfaces generated by a flexible cutting tool. Based on the measurement of the roughness *Ra* in the cut trace at any measured depth *h* (mm), the new mechanical constant *K_plmat_* is determined. From this parameter, equivalents of mechanical material parameters in the elastic as well as the plastic areas of deformation are determined, including numerical and graphical parameters in the case of engineering and true *σ–ε* diagrams, including the determination of deformation limits and their prediction for various kinds of materials. The method of determination of equivalents of mechanical material parameters from the topography of surfaces generated with a flexible cutting tool enables the sufficiently accurate determination of mechanical equivalents of material parameters even in a contactless and non-destructive way.

### 4.1. Surface Topography Function

**Figure 3:**
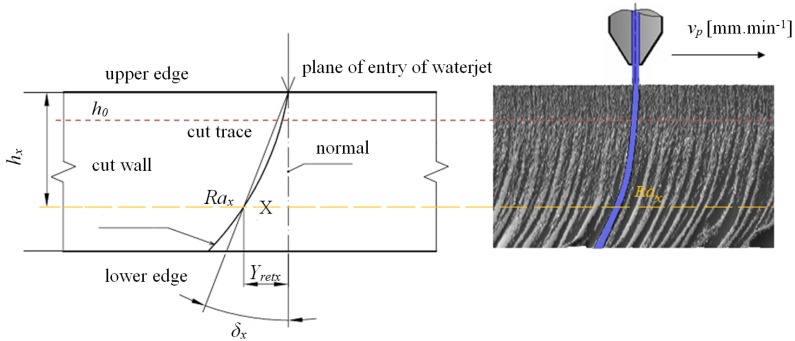
Geometrical parameters of the edge of cut in the case of abrasive waterjet cutting and method of determining parameters *Ra_x_*, *Y_retx_*, *δ_x_*, *h_x_* at point X in cut trace.

In this part of the derivation, the main geometrical parameters of the surface topology and their mutual functional and distribution relationships are defined. The knowledge of the topography function is of great importance for the analysis and prediction of the surface topography state for any change in the technological regime and/or change in the cut material [25,26]. Geometrical parameters provide information about changes in shape and dimensions of a permanently deformed body. It is the measurable permanent deformation on the cut wall surfaces that belongs to the most important geometrical parameters. Permanent deformations evaluate the process qualitatively (change in physical-mechanical properties) as well as quantitatively (change in outside dimensions). When studying the geometry of cuts, the continuous body, which is composed of spatially interconnected distributed points, is taken as the basis. The deformation of the continuous body is considered as a continuous mutual change in positions of individual points. Geometrical parameters (elements of permanent deformation) are divided into linear and angular values. Linear parameters express changes in linear dimensions of the whole body or its specific parts (grains, fibres). Angular parameters express changes in measured angles in the whole body or only in its specific parts. To assess the total deformation of the continuous body, the geometry of the cut wall surface after abrasive waterjet cutting is examined. As the main geometrical elements of the cut wall surface, the following parameters are proposed: surface roughness *Ra*, cut trace retardation *Y_ret_*, angle of curvature of cut trace (deviation) *δ* and depth of cut *h_cut_*, and potentially the thickness of the sample being cut (Figure 3).

The surface roughness grows in all places where deformation stress and deformation force, *i.e.*, the working ability of the tool, decrease [27]. For the assessment of surface roughness as the permanent deformation, merely two geometrical states, *i.e.*, initial and final, have usually been considered so far. This is why it is necessary to proceed in short elementary time stages *dt*. A theoretical basis for the derivation of the topography function for selected main variables is the use of the stress-strain parameters of the cut material in conjunction with a solution for the mechanical equilibrium of the system: material properties–tool properties–deformation properties as seen from Equation (1).

(1) $$Ra=f(Re,\;v_{p},\;p,\;d_{0},d_{a},\;m_{a},\;L,Y_{ret},\;\;\delta ,\;h_{cut}\ldots )$$

The previous equation contains the following parameters: *h_cut_*—depth of cut (mm), *Re*—yield point (MPa), *Y_ret_*—retardation of the cut trace (mm), *δ*—angle of curvature of cut trace (°), *v_p_*—traverse speed of the cutting head (mm·min^−1^), *p*—pressure (MPa), *d_0_*—orifice diameter (mm), *d_a_*—focusing tube diameter (mm), *m_a_*—abrasive mass flow rate (kg·min^−1^) and *L*—nozzle-material surface distance (mm). It is a set of both material and main technology parameters for machining the test samples.

When a beam is subjected to stress, the existence of a neutral plane is found. By analogy, the zone which we have named the neutral plane *h_0_* is one of the zones in flexible cuts. Here, the tensile and compressive states of stress are aligned. Location of the plane *h_0_* in cuts varies depending on the material. The position of neutral plane *h_0_* is readable e.g., in Figure 3, in this article, where it significantly separates the smooth surface from the grooved one.

From the measured data, their analysis and interpretation, we defined a neutral plane at the local minimum of the topography function at the roughness value *Ra* = *Ra_0_* = 3.7 μm. *Ra_0_* is an empirically determined value obtained from the surface topography measurements of materials. The value obtained by calculation is 3.7 for different materials. Its range of fluctuation is between 5% and 10% depending on the type of measurement selected [28]. For example, for an optical profilometer, it is 5%, for surface roughness tester SJ 401, it is 10%. These deviations are given by a principles of measurement and their accuracy. The determination of both, *i.e.*, the geometric parameters and the position of equilibrium plane (*Ra_0_*, *h_0_*) in the flexible cut, is an important analytical factor. The term “tool properties” can be replaced by the term “technological properties”. The whole set of properties, physical, mechanical and technological, is in close connection with and affects the mechanism of surface disintegration. A serious technological factor in material machining is the index of material machinability. This is an indicator for the suitability to use a specific set of technological machining parameters. To improve the properties of abrasive waterjet cutting technology, it is necessary to introduce a mathematical approach to the assessment of material machinability limits and classes. For this reason, the original “plasticity” factor *K_plmat_* is introduced. The parameter *K_plmat_* is based on the direct measurement of selected geometrical elements at any point X on the surface of cut wall according to the illustration in Figure 3. It is a comprehensive and empirical material parameter given in physical units (µm) that satisfies Equation (2) and is of high importance to other analyses of the process [21].

(2) $$K_{plmat}=\frac{Ra\cdot h_{cut}}{Y_{ret}}$$

Moreover, the parameter *K_plmat_* provides a direct connection to the elastic-strength properties of cut materials and to the laws of classical elasticity and strength, because a relation between the parameter and Young’s modulus in tension *E_mat_* in Equation (3) is also valid with sufficiently verified closeness [21].

(3) $$K_{plmat}=\frac{{\text{10}}^{\text{12}}}{E_{mat}^{\text{2}}}$$

The value 10^12^ is only the proportionality constant unit to get *K_plmat_* in µm. Hence, the following explicit equations for the dependence of the main deformation parameters Equations (4)–(7) on the depth of cut *h* for irregularities of the surface characterised by parameters *Ra*—roughness, *Y_ret_*—retardation of cut trace, *h*—instantaneous depth and *δ*—deviation angle can be stated:

(4) $$Ra=\frac{10^{12}\cdot Y_{ret}}{E_{mat}^{2}\cdot h_{cut}}$$

(5) $$Y_{ret}=\frac{E_{mat}^{2}\cdot Ra\cdot h_{cut}}{10^{12}}$$

(6) $$h_{cut}=\frac{10^{12}\cdot Y_{ret}}{E_{mat}^{2}\cdot Ra}$$

(7) $$\delta =arctg\left(\frac{Y_{ret}}{h_{cut}}\right)$$

**Figure 4:**
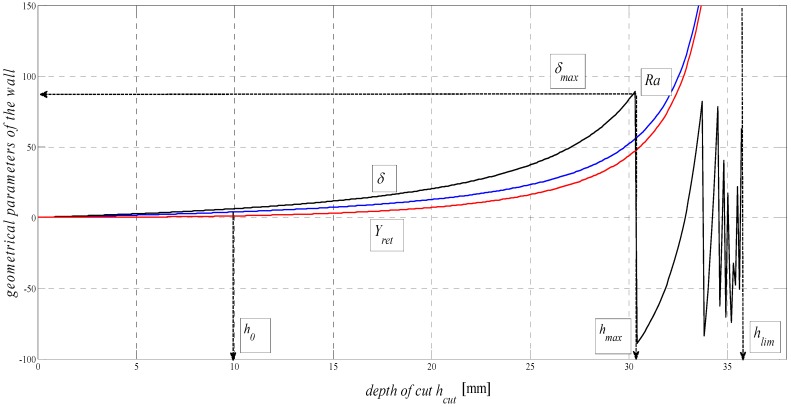
Dependence of main deformation parameters of the surface in the plane containing the cut trace on depth (*Ra*, *Y_ret_*, *δ*) = f (*h_cut_*) for steel AISI 309 material, *E_mat_* = 167.2 GPa.

According to the derived equations, in Figure 4, there are relationships concerning the distribution of geometrical elements of surface topography in the plane containing the cut trace. The graphic dependence *δ* = f (*h*) documents the achievable depth of the cut *h_lim_* in a specific material and the decomposition of the cut trace after reaching the limit angle of deviation *δ* = 90°. The limit theoretical depth can be expressed by the relationship *h_lim_* = 10^3^·*K_plmat_* (mm), which corresponds to the result predicted by theory, as well as the results of the statistical evaluation of the experiments suggested.

### 4.2. Areas and Limits in the Process of Elastic-Plastic Deformation of a Material

**Figure 5:**
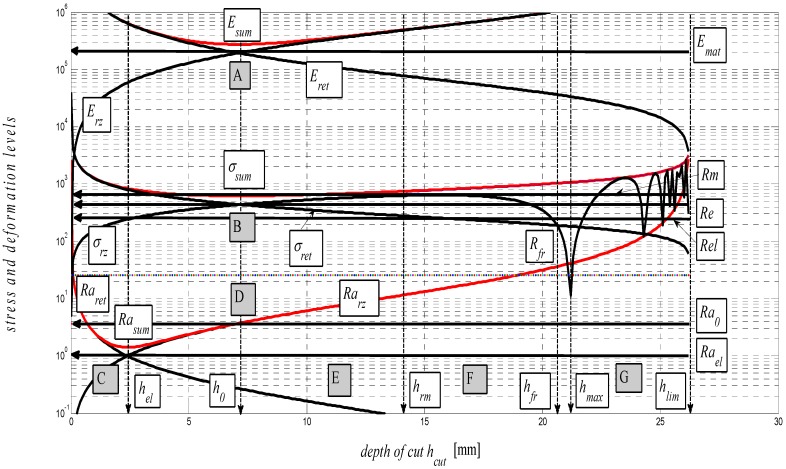
Stress and deformation levels of steel AISI 304 material, *E_mat_* = 195 GPa.

The graph in Figure 5 represents a complex mathematical model of the analysis of stress-strain functions under external stress on a material in the cut with a flexible tool. In particular, it is the topographic state of surface after cutting with a hydroabrasive tool (AWJ). The authors have been dealing with studying the topography of surfaces after cutting with AWJ and laser tools for many years. Strain traces remaining after the use of the flexible tools, namely, directly react to the mechanical resistance of the material under stress according to the shape of topography of the separating cut. Mechanical and topographical components are well measurable and allow for feedback control. Moreover, the whole process can be mathematically modelled. Since we know and have already analytically defined all important features relating to the surface topography and their relationship to the mechanical state of the core of materials, the whole process of deformability may be predicted in an analytical way and, thus, without any need for making cuts. The diagram of *σ*-*h_cut_*, or *σ*-*ɛ* or timing of *σ*-*t_cut_* may be assessed precisely at individual tensometric limits or at any point, and continuously using continuous functions, or discreetly as well. Especially, a precise identification of the elastic limit *Rel*, yield strength *Re* as well as the Young’s modulus *E_mat_* is a problem in classic tensile tests and for certain materials. At the same time, they are very important material parameters, which represent variables required for stability equations in designing machinery, buildings and structures. In case of AWJ, it is a cold cut not affected by high temperature, surface melting, *etc.* as in the case of flexible, disintegrating tools like laser, oxygen, or plasma.

In theory, the method is based on newly derived equations of equilibrium between main variables for surface geometry and for the material itself. The topic of solving the process of deformability led the authors to develop analytical constructions for the overall expression of tensometric states of material induced by external stress. It is actually a set of tensometric states linked to each other in time according to certain laws. The issue falls within the theory and practice of elasticity and strength. However, in available professional documentation, none of the issues are resolved comprehensively, or are completely grounded. The reason for this state consists in continuing problems with theoretical resolving of the transition from ideally elastic to quasi-elastic and plastic stress-strain states. So, in discussions on this particular topic, often professionally ambiguous attitudes prevail. The following symbols used in Figure 5 are: *E_sum_*—overall Young’s modulus (MPa), *E_ret_*—component of *E_mat_* for tension (MPa), *E_rz_*—component of *E_mat_* for compression (MPa), *σ_sum_*—total stress (MPa), *σ_rz_*—compressive component of stress (MPa), *σ_ret_*—tensile component of stress (MPa), *Ra_sum_*—total surface roughness (µm), *Ra_rz_*—compressive component of surface roughness formation (µm), *Ra_ret_*—tensile component of surface roughness formation (µm), *Rm*—conventional ultimate tensile strength (MPa), *h_rm_*—depth at ultimate strength (mm), *h_fr_*—depth at failure (mm), *h_max_*—maximal depth (mm), *Ra_el_*—roughness at elastic limit (µm), *Re_el_*—elastic limit (MPa), *R_fr_*—ultimate strength at failure (MPa).

**Figure 6:**
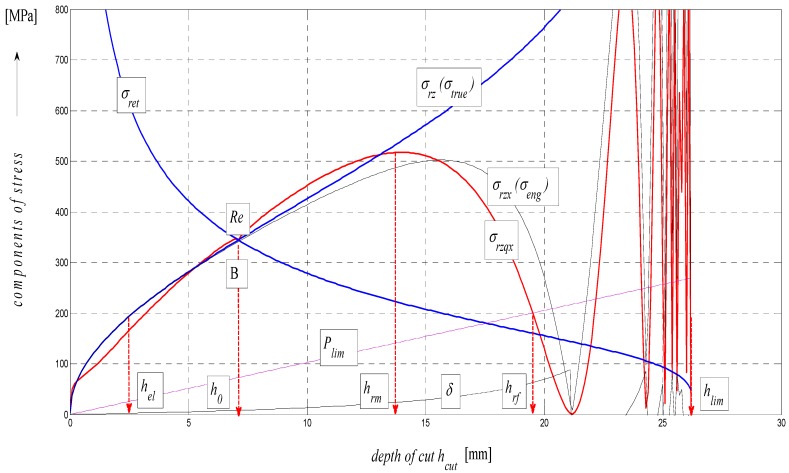
Components of steel AISI 304 material, *E_mat_* = 195 GPa.

The analytical construction of the graph in Figure 5 is made on the basis of the interpretation of initial topography values measured using a photodetector and values from tensile tests on a test-piece made of steel AISI 304. Except the original data from a topographical trace after applying a disintegration tool, a series of static and continuous parameters can be calculated using the values of *RMS* and *Ra* for determining the instantaneous tensometric state of material. For this purpose, the system of equations is used, which creates an algorithm in accordance with patent applications submitted under the registration numbers [21]. Recently, the possibility to generalize the Hooke’s law into a plastic area derived from the distribution of the roughness parameter *Ra* and other elements of the surface geometry during the deformation of material in a way in accordance with the mentioned patent applications has been analytically used. We are now coming to the family of curves listed in the legend to Figure 5 and Figure 6 which creates a comprehensive picture of the stress-strain or tensometric functions for a particular type of material. The amplitudes of the functions are expressed in a logarithmic scale on the Y-axis. On the X-axis, a depth of cut *h_cut_* is introduced. The depth of cut on the X-axis may be replaced with the relative longitudinal deformation *ε*, or also the time *t_cut_* according to the relations derived by authors [21]. For the elastic area C and *h_cut_* ˂ *h_el_*, *ε_el_* is applied according to Hooke’s law, and *ε_pl_* according to Equation (24) it is applied for depths *h_cut_* ˃ *h_el_*.

Three tensometric levels are distinguished: modulus A, stress B and strain C. At those levels, the decomposition into compression and tension components is performed. At the modulus level, the constant value of Young’s modulus, modulus load (compression) component *E_rz_*, modulus deformation (tensile) component *E_ret_*, and the cumulative component *E_sum_* may be seen. The same is true by analogy for the levels B and C, and also for the retardation components of cut trace *Y_ret_* and their intersection D. The roughness components in the node C set out the depth level *h_el_* at the elastic limit *Rel*. The nodes A, B and D define the position of the neutral plane *h_0_* at the yield point *Re*. To the depth level *h_0_*, the *h_0_*-*h_rm_* section is tied at the ultimate strength *Rm* marked with the letter E and outlines the area of elastic-plastic deformations. The *h_rm_*–*h_max_* section marked with the letter F outlines the area of plastic deformations and the section G the loss of original structure and cohesion up to the plastic creep of the material. For a clearer illustration, in Figure 6, also detail of the tension node B in Figure 5 in linear coordinates is shown.

### 4.3. Intensity of Increase in Plasticity of Material in the Process of Deformation

The functions for an analytical expression of the intensity of an increase in plasticity are those that affect the deformation intensity of trace and the surface of separating cut wall. According to Figure 5, those functions are growing adequately with the growth of the angle *δ* and the plasticity index *INDpl* and depending on the depth *h*, or in accordance with the relative deformation *ε* or according to the time *t_cut_*. In the default expression, the notation of functional relations in the form of *INDpl* = f (*E_mat_*, *E_rz_*, *σ_rz_*, *K_plmat_*, *Ra*, *h_cut_*, *h_0_*, *ε*, *δ*, *δ_0_*, *t_cut_*…*σ_res_*) can be used. Also, a significant influence of the growth of residual *σ_res_* on the increase in plasticity of the surface and the core of material is expected. The behaviour of the function *σ_res_* is given, according to the derivation by the authors [29] by the relationship Equation (8)

(8) $$\sigma_{res}=\sigma_{rz}\cdot \sin{\left(\delta \cdot \frac{\pi }{180}\right)}^{2}$$

*σ_res_* = f (sin *δ*) is here, which justifies the influence of residual stress on the intensity of increasing plasticity. Therefore, it is also illustrated in the graph in Figure 5. The equation for *σ_res_* was verified using an ultrasonic method [29].

**Figure 7:**
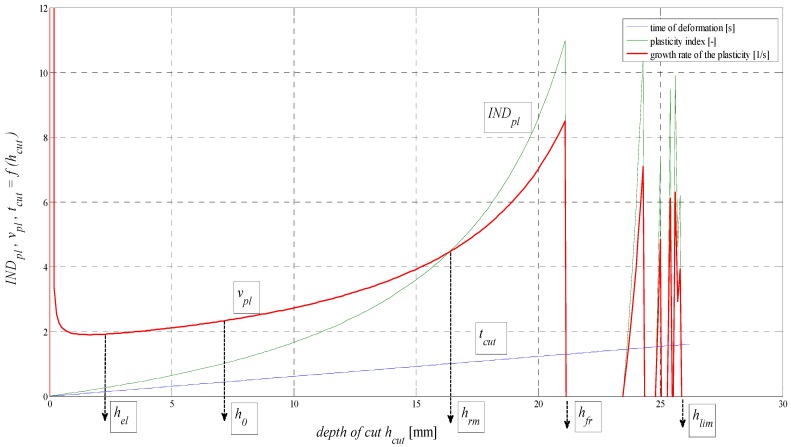
Analysis of f (*IND_pl_*, *v_pl_*, *t_cut_*) for AISI 304 material, *E_mat_* = 195 GPa.

The examination of plasticity rate *v_pl_* = *IND_pl_*/*t_cut_* (1/s) is also of interest. As shown in Figure 7, the local maximum *v_pl_* is within the first millimetre of depth where the disintegrating tool starts to penetrate into the material. 

Here, it concerns the point indentation stress and volumetric stress. After overcoming the elastic limits at *h_el_*, where *v_pl_* is a local minimum, it continues to grow according to depth. The deformation time tcut was derived by means of regression according to FFT. With good correlation conformity, it was confronted with the results according to [16]. Here, strain rates and the duration of disintegration behaviour in the AWJ cut were measured by means of the so-called visualization method. The equation for timing, according to the authors of the present article, can be found according to Equation (9) of

(9) $$t_{cut}=\frac{h_{cut}-0.10002}{13.9392}\cdot \sqrt{\frac{35.7707}{K_{plmat}}}$$

and is true, in general, for different materials defined here by the constant of plasticity, or cuttability *K_plmat_*. The parameter *INDpl* can be well utilized for the construction of deformation diagrams *σ_pl_* = f (*h*, *ε*, *t_cut_*) where *σ_pl_* = f (*INDpl*). Similarly, for the calculation of deformation stress to create surface roughness, the relationships in Equations (10) and (11) derived by authors apply.

(10) $$\sigma_{ra}=\frac{10^{-3}\cdot Ra\cdot E_{mat}}{Ra_{0}}$$

(11) $$\sigma_{rax}=\frac{\sigma_{ra}}{\cos\text{​ }\delta }$$

After completion of the basic notation, the equations of a specified approximation form are obtained according to Equations (12) and (13):

(12) $$\sigma_{pl}=\frac{0.2637\cdot 10^{-3}\cdot IND_{pl}\cdot E_{mat}\cdot \left(\frac{h_{0}}{h_{cut}}\right)\cdot \sqrt{\frac{P_{\lim}\cdot 4.94}{\sqrt{0.5}}}+\sqrt{E_{rz}}}{2}$$

(13) $$\sigma_{plx}=\sigma_{pl}\cdot \cos\text{ ​}\delta^{{\left(\frac{h_{0}}{h_{cut}}\right)}^{4}}$$

Here, the parameter *P_lim_* in Equation (14) represents a stress development in the core of the material in (MPa).

(14) $$P_{lim}=\frac{\sigma_{Re}}{K_{plmat}}\cdot h_{cut}\cdot {\left(\frac{20}{K_{plmat}}\right)}^{0.75}$$

**Figure 8:**
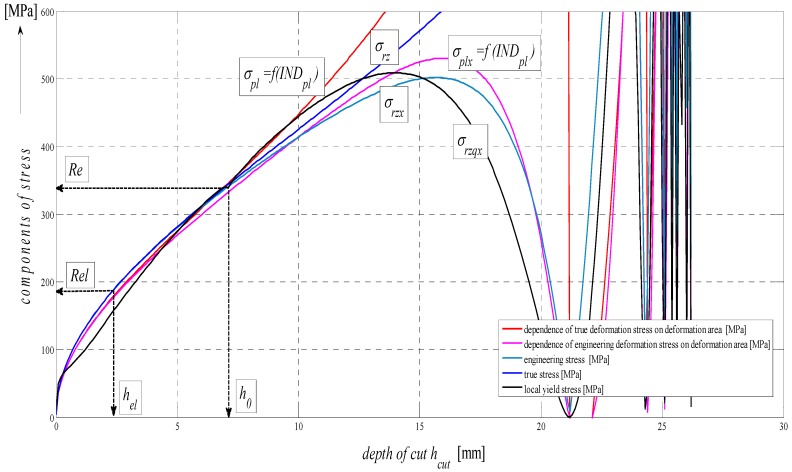
Analysis of components of stress on depth of cut for AISI 304 material, *E_mat_* = 195 GPa.

A good comparative match with other functions *σ_rz_* and *σ_rzq_* for the calculation of deformation stress can be seen in Figure 8. From the composition of variables for the calculation of *σ_pl_*, it is also apparent that discrete values and individual limits in any depth *h*, thus *ε* or *t_cut_* can be read from the graph. Substituting the discrete values, the deformational diagrams *σ_pl_* = f (*h*, *ε*, *t_cut_*) can thus be constructed by means of a discrete point construction as well. As grounded above, the analysis of the deformability process may be carried out, and can be predicted purely analytically and, thus, without any need for making test cuts.

**Figure 9:**
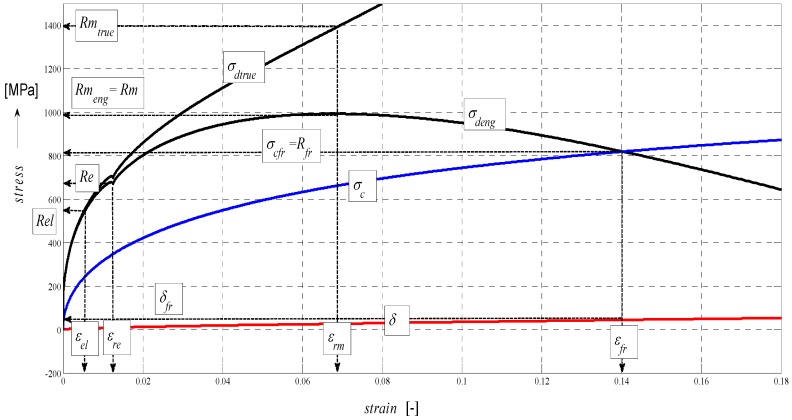
Diagram for STN 15230 steel, dependence of functions on relative elongation.

The basic considerations of the concept of analytical processing can be illustrated most simply by geometrical similarity in the curves of integral (total) stress in the modulus area (A), and more specifically, those of development of stress in the material in the stress area (B) and those of development of surface roughness in the deformation area (C) according to a diagram in Figure 9. This similarity is hypothetical and forms a theoretical basis for the derivation of required relations. The mechanism of actual development of disintegration and deformation processes corresponds well with the distribution of stresses and deformations in the studied cut, providing, thus, information about the occurrence and development of tensile and compressive stresses and the deformations induced by them.

Based on the Equations (3)–(7) derived for the basic form of a topography function for the plane containing the trace, functional relations of stress and deformation quantities to instantaneous surface deformations in the cut can be defined. Thus, the following equations for the decomposition of these quantities into the tensile and compressive components, including their summarized values and values on the level of neutral plane will be obtained. For the total stress, Equation (15), which contains the decomposition component of stress *σ_rz,_* of Equation (16) for compression and the decomposition retardation component of tension *σ_ret_* of Equation (17), applies.

(15) $$\sigma_{sum}=\sqrt{(\sigma_{rz}^{2}+\sigma_{ret}^{2}})$$

(16) $$\sigma_{rz}=\sqrt{E_{rz}}$$

(17) $$\sigma_{ret}=\sqrt{E_{ret}}$$

In the equations, the following parameters are included: Young’s modulus in tension *E_mat_*, decomposition component of *E_mat_* for compression *E_rz_*, decomposition component for tension *E_ret_* and instantaneous surface plasticity *K_pl_,* which is defined as instantaneous deformation area given by the product of instantaneous values of roughness *Ra* and those of depth *h*. Then, the equations for the calculation of compressive stress in the cut can be generally written in the form of Equation (18):

(18) $$\sigma_{rz}=\sqrt{E_{rz}}=\sqrt{E_{mat}}\cdot \sqrt[{4}]{\frac{K_{pl}}{K_{plmat}}}$$

where instantaneous surface plasticity is defined by Equations (19) and (20)

(19) $$K_{pl}=Ra\cdot h_{cut}$$

(20) $$E_{rz}=E_{mat}\cdot \sqrt[{4}]{\frac{K_{pl}}{K_{plmat}}}$$

and for the dissipation component of stress, the following relation holds true as per Equation (21)

(21) $$\sigma_{rzx}=\sigma_{rz}\cdot \cos\delta$$

where the member cos *δ* is a dissipation factor in the cutting technologies utilizing flexible tools. For a sharp and rigid tool, such as a non-blunt turning tool, where *δ* = 0, it would be true that cos *δ* = 1 and *σ_rzx_* = *σ_rz_.* It holds for the engineering curve Equation (22) and for the true one as per Equation (23)

(22) $$\sigma_{rzx}=\sigma_{deng}$$

(23) $$\sigma_{rzxy}=\sigma_{true}=\frac{\sigma_{rz}}{\cos\delta }$$

To get the topography function, its onset in the initiation zone and the curvature of cut trace, the equations for the topography function in the plane that contains the trace have to be supplemented by solving the equation for that in the radial plane, *i.e.*, in the plane below the point of application of the tool radially to the surface of the sample, where roughness is usually measured in practice, is checked and then stated in tables.

### 4.4. Calculation Relations

Based on the input parameter *E_mat_* of the given material, it is especially a case of calculation of the plasticity coefficient *K_plmat_* according to Equation (2). For the construction of stress-strain diagrams, functional relationships for stress-strain functions are essential. The deformation parameter *δ* can be used suitably for the expression of relative deformation or relative elongation in the form ε = δ/90(-). More specifically, the relative elongation can be expressed using all partial deformations of the surface in the form of Equation (24)

(24) $$\varepsilon =10^{3}\cdot \frac{Y_{ret}}{E_{mat}}$$

Then, it holds true for Equation (25)

(25) A=100⋅ε

The cos *δ* function represents a dissipation factor and is used for the division of stress into the deformation dissipation, *i.e.*, engineering component *σ_rzx_* according to Equation (22) and the non-dissipation, *i.e.*, actual component *σ_rz_* = *σ_true_* (actual stress), as follows from the measurement results. Equations for the expression of a relationship between stress and deformation and for the construction of stress-strain diagrams are derived in the form of Equations (26)–(29). As a matter of fact, the equations represent generalized Hooke’s law and hold true for the whole stress-deformation area and generally for all materials. For the theoretical branch of so-called actual stress, a relationship as per Equation (28) holds true; to the theoretical branch of so-called engineering stress, a relationship as per Equation (29) with reduction using the cos *δ* function applies.

(26) $$A=0.1\cdot \frac{Y_{ret}}{Y_{ret0}}\cdot \frac{h_{cut}}{h_{0}}\cdot \frac{Ra_{rad}}{Ra_{rad0}}$$

(27) $$\varepsilon^{n}=\varepsilon =\frac{A}{100}$$

(28) $$\sigma =\varepsilon \cdot E_{mat}$$

(29) $$\sigma_{x}=\varepsilon \cdot {\left(\cos\delta \right)}^{h_{0}}\cdot E_{mat}$$

For the auxiliary function of *M_taž_* (%) for the determination of the elongation limit, the relationship expressed in Equation (30) is valid:

(30) $$M_{taž}=\left(10^{-3}\cdot \frac{Ra_{0}\cdot \varepsilon }{\varepsilon_{0}}\cdot \cos\delta^{h_{0}}\right)$$

To the development of local roughness in the yield point region, the development of local stress *σ_rzq_* according to the relationship in Equation (31) is related, where *Ra_q_* is the local roughness for Equation (32) in the yield point region.

(31) $$\sigma_{rzq}=10^{-3}\cdot E_{mat}\cdot \frac{Ra_{q}}{Ra_{0}}\cos\delta^{h_{0}}$$

(32) $$R_{aq}=\left(\left(\log{\left(h\right)}^{2}+\log{\left({\left(h\cdot tg\;\delta \right)}^{-1}\right)}^{0.25}\right)\right)$$

The *σ_c_* function simulates stress in the material core; it means the permanent strength of the material, and determines ductility on the envelope of the stress-strain curves as per Equation (33)

(33) $$\sigma_{c}=\frac{E_{mat}^{0.5}}{K_{plmat}}\cdot h_{cut}$$

### 4.5. Construction of the σ–ε and σ-h Graphs

**Figure 10:**
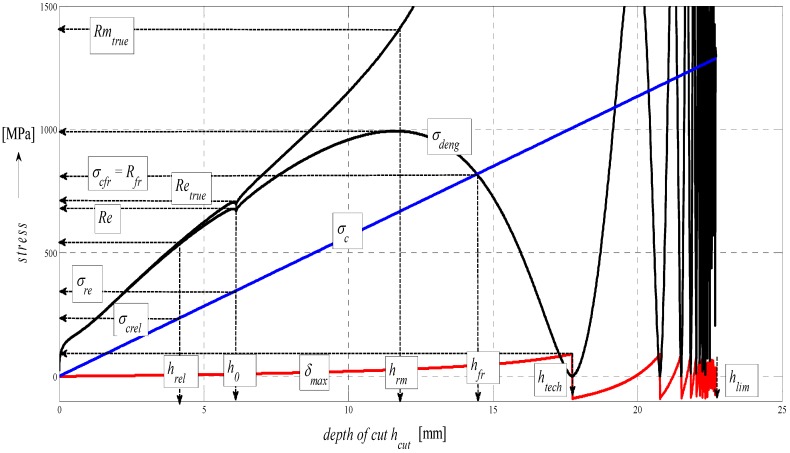
Diagram for STN 15230 steel, dependence of functions on absolute depth.

**Figure 11:**
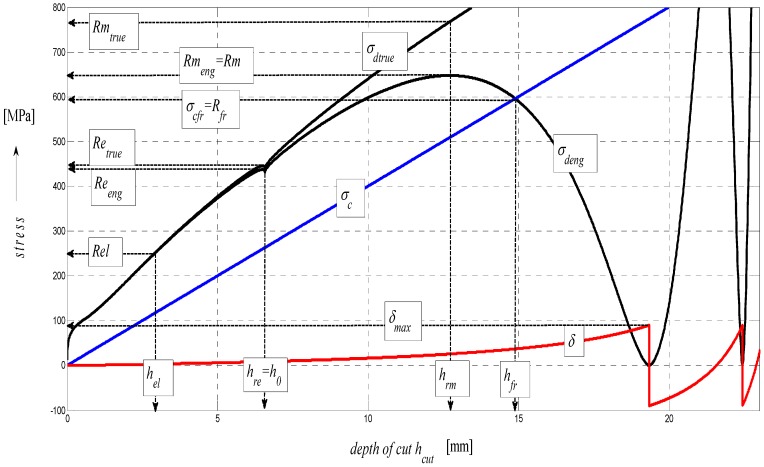
Diagram for EN S355J0 steel, dependence of functions on absolute depth.

**Figure 12:**
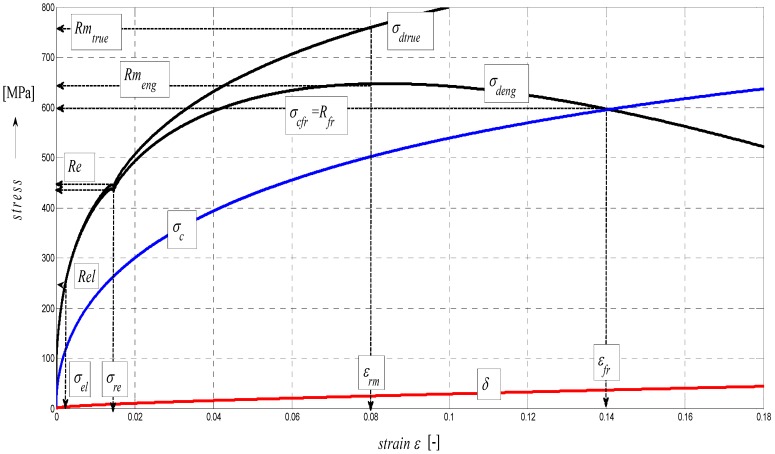
Diagram for ENS355J0 steel, dependence of functions on relative elongation.

**Figure 13:**
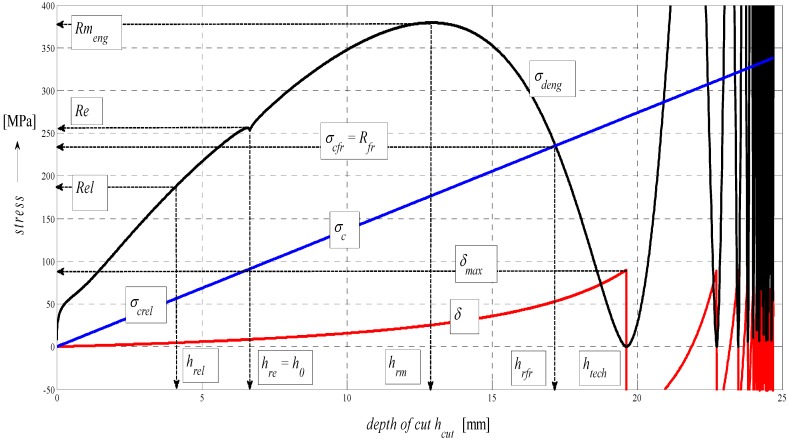
Diagram for AISI 1020 steel, dependence of functions on absolute depth.

**Figure 14:**
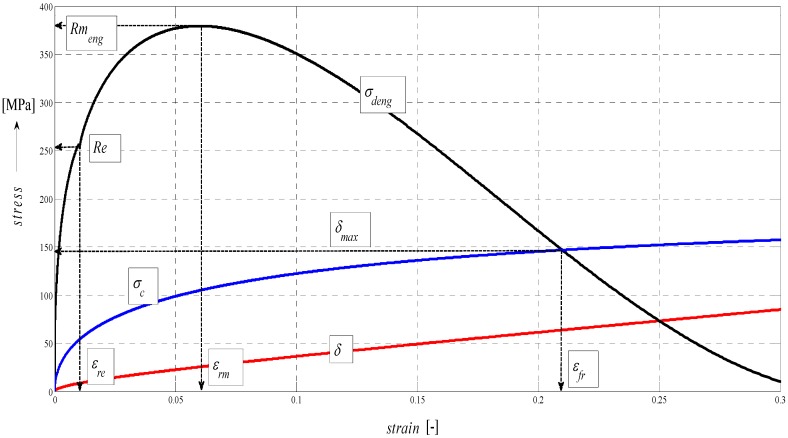
Diagram for AISI 1020 steel, dependence of functions on relative elongation.

**Figure 15:**
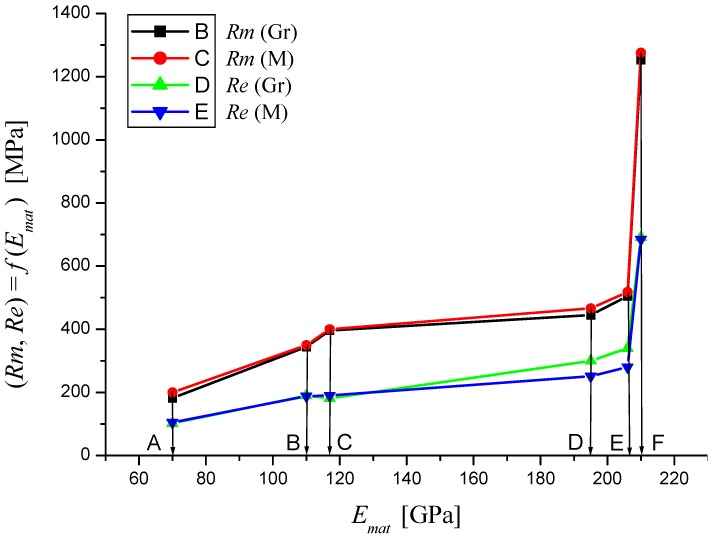
Dependencies of material parameters (Mes) and theoretical (Gr) for materials A—aluminium, B—titanium, C—commercially pure copper, D—AISI 304, E—commercially pure iron, F—AISI 309.

**Figure 16:**
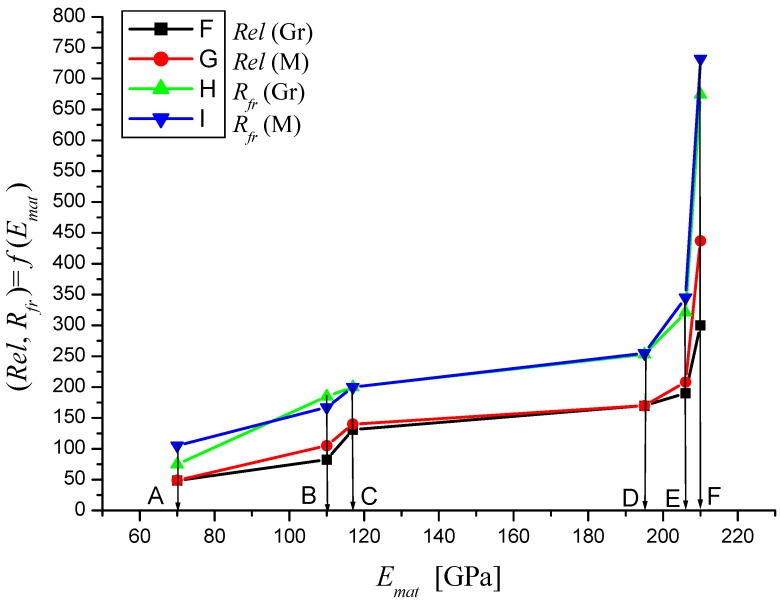
Dependencies of material parameters (Mes) and theoretical (Gr) for materials A—aluminium, B—titanium, C—commercially pure copper, D—AISI 304, E—commercially pure iron, F—AISI 309.

**Figure 17:**
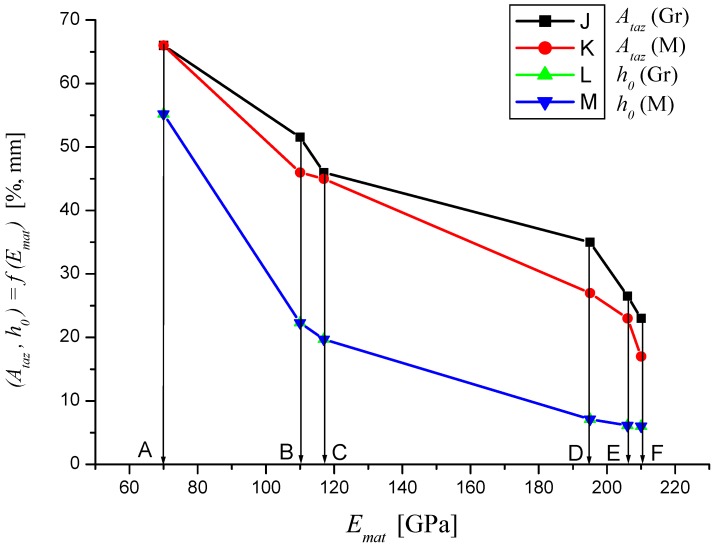
Dependencies of material parameters (Mes) and theoretical (Gr) for materials A—aluminium, B—titanium, C—commercially pure copper, D—AISI 304, E—commercially pure iron, F—AISI 309.

In the following figures, the dependence of stress-strain functions on relative longitudinal elongation (Figure 9, Figure 10, Figure 11, Figure 12, Figure 13 and Figure 14) is shown, as well as a comparison between conventional stresses for single materials in the case of measured values (Mes) and theoretical values (Gr (see Figure 15, Figure 16 and Figure 17), the difference between them being less than 10%. They include the following equivalents of basic tabular elastic-strength parameters of technical materials, including the values of the parameters at the main, arbitrarily chosen limits of deformability: *σ_c_*—stress in material core (MPa), *R_fr_*—ultimate strength at failure (MPa), *Re*—yield point (MPa), *Rel*—elastic limit (MPa), *A_taz_*—elongation limit (%), *δ_max_*—angle of deviation of cut trace for depth *h_max_* (°), *h_Re_*—depth at yield point (mm), *ε_el_*—relative elongation at elastic limit (-), *ε_Re_*—relative elongation at yield point (-), *ε_Rm_*—relative elongation at ultimate strength (-), *ε_fr_*—relative elongation at failure (-), *Rm_eng_*—engineering ultimate strength (MPa), *Rm_true_*—true ultimate strength (MPa), *σ_dtrue_*—true deformation stress (MPa), *σ_deng_*—engineering deformation stress (MPa).

The comparison of the stress-strain curves of the figures given above show that the tensile test performed in VÚHŽ Ltd. perfectly reproduces the experimental test. Thus, it shows that the equivalents of mechanical parameters and their relationships are correctly determined.

The equivalents of mechanical parameters obtained from the above given equations seem sufficient to give the response to the elastic and plastic zone, necking occurrence and fracture. To confirm our method, another experiment has been performed with different materials (see Table 1 and Table 2). This table contains the values obtained by tensile testing and the values obtained by theoretical prediction calculations (see equations above). All values can be also verified in [30].

**Table 1:** Values obtained by tensile testing and values obtained by theoretical prediction calculations.

Parameter	Titanium	Titanium	AISI 309	AISI 309	Aluminium	Aluminium
	Mes	Gr	Mes	Gr	Mes	Gr
*E_mat_* (MPa)	110000	110000	210000	210000	70000	70000
*K_plmat_* (μm)	82.64	82.64	22.68	22.68	204.08	204.08
*Rm* (MPa)	350	343.74	1275.55	1252.72	200	182
*Re* (MPa)	188	189.68	685.15	691.28	105	102
*Rel* (MPa)	120	82.31	437.33	299.97	49	48.59
*R_fr_* (MPa)	185	200.96	674.22	732.373	75	105
*A_taz_* (%)	26.5	27	51.55	17	23	23
*h_0_* (mm)	22.3	22.3	6.1	6.1	55.2	55.2
*ε_fr_* (-)	0.27	0.27	0.3	0.17	0.23	0.23

**Table 2:** Values obtained by tensile testing and values obtained by theoretical prediction calculations.

Parameter	AISI 304	AISI 304	Iron CP	Iron CP	Copper CP	Copper CP
	Mes	Gr	Mes	Gr	Mes	Gr
*E_mat_* (MPa)	195000	195000	206000	206000	117000	117000
*K_plmat_* (μm)	26.3	26.3	23.56	23.56	73.1	73.1
*Rm* (MPa)	505	518	445	466	400	396
*Re* (MPa)	340	280	300	251	240	238
*Rel* (MPa)	190	181	190	208	190	190
*R_fr_* (MPa)	200	187	345	321	255	253
*A_taz_* (%)	66	66	45	46	45	46
*h_0_* (mm)	7.1	7.1	6	6	19.7	19.7
*ε_fr_* (-)	0.66	0.66	0.45	0.46	0.45	0.46

## 5. Results and Discussion

Surface topography is important in determining how a material performs and how it affects the deformation behavior of a material. Therefore, it is logical to think that some relationships might exist between the surface topography and the material under load. In principle, the technological process of disintegration leaves its specific “signature” on the material surface properties; what is meant is technological heredity. The microstructure influences tensile properties such as the modulus of elasticity, the yield and tensile strength as well as elongation or time of deformation. The relationships between these properties are in fact complicated. A way to quantify such relationships is given in this paper by the diagrams indicated in Figure 9, Figure 10, Figure 11, Figure 12, Figure 13 and Figure 14. Surface roughness always increases where deformation stress and deformation force diminish, and accordingly, the working ability of a tool decreases. *K_plmat_* (μm) and *IND_pl_* (-) are new parameters that increase with the plasticity of the cut material, where *K_plmat_* is determined from three deformation parameters according to Figure 3. The authors have found that the influence of the Young´s modulus is unambiguously shown on the main topographic parameters of the final surface of cutting walls, that is: roughness of surface *Ra* in the trace of cut and *Ra_d_* in the cross section, the deviation of the cut trace from vertical plane *Y_ret_*, the deviation angle of the cut trace from vertical plane *δ* and the achieved depth of cut *h or* *ε*, or *t_cut_*. Another important analytical factor is the determination of position of equilibrium/neutral plane *h_0_* in the cut produced by abrasive waterjet cutting. The depth level of the plane is illustrated in Figure 3, Figure 6, Figure 7, Figure 8, Figure 11 and Figure 13. It forms the marked boundary of a relatively smooth cut above the level *h_0_*. Below the level *h_0_*, the curvature of trace and roughness grows vigorously. The explanation of this phenomenon can be seen in the equalization of tensile and compressive stresses in the cut. The value *h_0_* varies for different materials and is a function of *K_plmat_* and *E_mat_*. This unique point *h_0_* then designates the transition from the previous, almost ideal elastic behavior to the subsequent behavior approaching perfect plastic flow. Stages in *σ–ε* and *σ–h* diagrams are associated with the changes in surface topography (deformation) of the material. Therefore, knowing these stages is relevant for the current description of the deformation behavior of the material. In order to confirm the correctness of the theory, the results were verified by conducting a tensile test in the certified VÚHŽ laboratory (VÚHŽ, Dobrá, Czech Republic). The numerical values, graphical outputs and load diagrams, *σ–ε* and *σ*-*h* respectively, according to the new method of determination may supplement the laboratory data and comprehensively reflect the real characteristics of each structural material.

## 6. Conclusions

The surface roughness of materials processed by abrasive waterjet cutting technology has been studied, and the mechanical equivalents from the surface topography were derived. The changes in surface texture were competitively used by a fundamentally new method of determination of equivalents of mechanical parameters of materials generated with a flexible cutting tool. Based on the results presented in this study, we were able to identify and derive the following:

- equilibrium of deformation functions of the surface topography,
- deformation capacity, plasticity coefficient *K_plmat_* and *IND_p_*_l_ and their relations to *E_mat_*,
- equations for the elements of surface topography in the trace of cuts and in the radial plane,
- methods of solving the stress-strain functions according to surface topography,
- construction of equivalents to the *σ*-*h* and *σ–ε* diagrams from the parameters of cut.

In conclusion, it can be stated that merely material basic data is presented in this study, *i.e.*, the data that is read directly from diagrams *σ–ε* and the data that determines the principal stress-strain limits and can be compared with the measured data or data from the tensile test. We determined the parameters *Rel*, *Re*, *Rm*, *R_fr_*, *A_taz_* and the relative deformation at individual limits. They are technically the most important strength limits in the diagrams of deformation of structural materials. With the strength limits, the deformation limits are adequately connected. Knowledge of the material parameters is required for the proper sizing of all buildings and structures in the work of designers. If we know the patterns of distribution of major topographical features, then we are able to express and predict the limits analytically. Thus, the presented method enables us to determine the mentioned strength and deformation limits of materials based on the derived proprietary algorithm. This algorithm and also the method of calculation of all main cutting parameters of the AWJ apparatus are part of the patent [24].

Based on these parameters and their relationships with the Young’s modulus *E_mat_*, a number of other equivalents of material parameters for comprehensive characterization can be derived from the elasticity–strength relationships and physics. For the engineering exploitation of the material, it is believed to be important to provide analytical relationships verified by experimental testing. This allows a simple consideration of structural alterations in the loaded surface and core of the material as a physico-mechanical continuum, which affects its stiffness and load bearing capacity and deformability. Load diagrams *σ–ε* and their numerical and graphical parameters according to the new method of determination supplement laboratory values and express the real characteristics of each structural material more comprehensively.
